# Anticoagulation Options for Coronavirus Disease 2019 (COVID-19)-Induced Coagulopathy

**DOI:** 10.7759/cureus.8150

**Published:** 2020-05-16

**Authors:** Alla Turshudzhyan

**Affiliations:** 1 Internal Medicine, University of Connecticut Health Center, Farmington, USA

**Keywords:** unfractionated heparin, low molecular weight heparin, covid-19, doac, nafamostat, d-dimer, fibrinogen, platelet count

## Abstract

As the coronavirus disease 2019 (COVID-19) pandemic is evolving, coagulopathy induced by the disease and its severe complications are raising concerns in the medical community. Because coagulopathy caused by COVID-19 has been difficult to control, it is important to have a better understanding of what therapies have been studied thus far and what therapies have demonstrated better outcomes for hospitalized patients. This review is focused on literature, research, and expert clinical judgments published in 2020 with a few references to articles published earlier. The review introduces the interim guidelines of the International Society of Thrombosis and Haemostasis (ISTH) for management of COVID-19-induced coagulopathy, discusses the efficacy of these guidelines in clinical settings, and summarizes the response of the scientific community to these guidelines and their clinical implications. Due to the failure of patients to respond to the prophylactic doses of heparin recommended by ISTH, higher doses of heparin may be necessary to achieve adequate anticoagulation. Patients’ resistance to prophylactic doses of heparin could be due to low levels of anti-thrombin and high levels of fibrinogen, which would reinforce the use of therapeutic doses of heparin in the early stages of hospitalization. The review also compares low-molecular-weight heparin (LMWH) and unfractionated heparin (UFH) as anticoagulant choices for COVID-19 patients. Given the complications specific to COVID-19, UFH may be a better choice of anticoagulant. Outpatient anticoagulation options are also reviewed. Changing qualified patients from vitamin K antagonists (VKA) to direct-acting oral anticoagulant (DOAC) for the convenience of less frequent monitoring may be appropriate. New anticoagulant, nafamostat, used in Japan is also discussed as a possible potentiate for heparin therapy.

## Introduction and background

The first cases of coronavirus disease 2019 (COVID-19) infection were identified and described in December of 2019 in Wuhan, China [1]. Since then, the disease has spread causing an overwhelming number of cases worldwide. The symptoms caused by COVID-19 range from mild upper respiratory symptoms to multiorgan failure complicated by severe hypercoagulable state. This coagulopathy has been observed in many severe COVID-19 cases and has been associated with poor prognostic outcomes [1,2]. This is why it is so important to have a good understanding of what causes the hypercoagulable state and how to best treat it. A deeper understanding of this will help guide the treatment and help prevent complications and poor outcomes related to the hypercoagulable state. This review will focus on the literature about management of coagulopathy in COVID-19 published in the year of 2020 with a few references to articles published earlier.

## Review

One of the important sets of interim guidelines for the management of coagulopathy in COVID-19 patients was introduced by the International Society of Thrombosis and Haemostasis (ISTH) on March 25, 2020 [3]. These guidelines recommend a prophylactic dose of low-molecular-weight heparin (LMWH) to be given to all COVID-19 patients requiring hospitalization, in the absence of any contraindications such as active bleeding and platelet count less than 25 x 10^9^/L [3]. The benefit of LMWH use in sepsis-induced coagulopathy was recently studied by Thachil et al. who found that LMWH appears to be associated with better prognosis in relation to mortality (40.0% vs 64.2%, p=0.029) [3]. In addition, LMWH has been shown to have anti-inflammatory properties which may help in the setting of elevated cytokines and proinflammatory state of COVID-19 cases [1].

One of the most common laboratory findings for patients requiring hospitalization has been an elevated D-dimer [2]. Tang et al. have identified that markedly elevated D-Dimer is a mortality predictor and if increased three- to four-fold, the patient should be considered for hospitalization even if asymptomatic as elevated D-dimer increases thrombin generation [2]. The other three laboratory markers that ISTH recommends measuring in patients with COVID-19 are fibrinogen, prothrombin time, and platelet count to monitor the development of disseminated intravascular coagulation (DIC) [3]. Of note, Tang et al. observed that in patients who did not survive the infection, 71.4% developed DIC on day four of admission while only 0.6% of surviving patients developed DIC during the same period [2]. This is why monitoring these laboratory values is essential when evaluating prognosis.

On March 28, 2020, soon after the ISTH came up with the interim guidelines, the Chinese Journal of Hematology published a case series on severe complications from the COVID-19 hypercoagulable state [4]. Seven cases were described where acrocyanosis developed as a complication of coagulopathy [4]. Six out of seven patients were initiated and continued on anticoagulation with a therapeutic dose of LMWH for a median duration of eight days with no improvement in limb ischemia [4].

On March 27, 2020, Tang et al. published a retrospective analysis of 449 patients’ 28-day mortality among heparin users and nonusers [5]. They found that 28-day mortality was lower for patients with sepsis-induced coagulopathy (SIC) score greater than or equal to four (40% vs 64.2%, p=0.029) or D-dimer greater than six-fold of upper limit of normal (32.8% vs. 52.4%, p=0.017) [5]. This study concluded that anticoagulation with seven or more days of therapeutic doses of heparin is associated with better prognosis in patients who have severe infection, meet SIC criteria, or have markedly elevated D-dimer [5].

Yin et al. followed up with another retrospective study published on April 3, 2020 comparing severe pneumonia induced coagulopathy by severe acute respiratory syndrome coronavirus 2 (SARS-CoV2) and non-SARS-CoV2 [6]. Four hundred forty-nine SARS-CoV2 patients and 104 non-SARS-CoV2 patients were retrospectively studied [6]. Patients with severe pneumonia caused by SARS-CoV2 had a higher platelet count than patients with pneumonia caused by non- SARS-CoV2 [6]. No difference on the 28-day mortality was established between heparin and non-heparin groups in SARS-CoV2 patients (30.3% vs. 29.7%, p=0.910) and in non-SARS-CoV2 patients (13.6% vs. 15.9%, p=0.798) [6]. They also observed that only SARS-CoV2 patients with six-fold elevation in D-dimer benefitted from therapeutic doses of anticoagulation [6]. There was no difference in mortality when stratified by D-dimer in the non-SARS-CoV2 patient group [6].

Multiple studies thus far have shown that heparin is beneficial in the treatment of COVID-19-induced coagulopathy, however, given some failures in patients’ response to treatment, it is important to establish the correct dose of heparin. Thachil et al. in The Versatile Heparin In COVID-19 published on April 2, 2020, raised questions about possibly needing a higher dose of LMWH in individuals with high body mass index, extremely high D-dimers, acute respiratory distress syndrome (ARDS), or increasing oxygen requirements [7].

On April 11, 2020, the Swiss Society of Hematology published recommendations that all in-hospital COVID-19 patients should receive thromboprophylaxis, which echoed the ISTH guidelines published earlier [8]. The recommendations, however, offered dose adjustments based on the patient’s weight, D-dimer levels as well as the development of renal, hepatic, or respiratory failure [8]. For patients with a creatinine clearance of greater than 30 ml/min, LMWH should be given, with an increase in the dose if the patient’s weight is greater than 100 kg [8]. For patients with a creatinine clearance of less than 30 ml/min, unfractionated heparin (UFH) should be given, with an increased dose for patients weighing more than 100 kg [8]. They also suggested increasing anticoagulation to therapeutic ranges for patients whose D-dimer is significantly elevated or there is a concern of hepatic, renal, or respiratory failure [8]. They did not elaborate on the use of oral anticoagulants at that time due to a lack of data [8].

The use of oral anticoagulants and their application in the treatment of COVID-19 patients was further explained by Thachil et al. in DOACs and ‘Newer’ Hemophelia Therapies in COVID-19 published on April 13, 2020 [9]. They suggested that it would be appropriate to transition patients currently on vitamin K antagonists (VKA) to a direct-acting oral anticoagulant (DOAC) to reduce the number of needed laboratory testing [9]. This would not apply to patients with mechanical valves or antiphospholipid syndrome [9]. The need to transition from VKA to DOAC in the outpatient setting for patients without contraindications due to lack of access to frequent international normalized ratio (INR) monitoring during the pandemic was also echoed by Hermans et al. in their Impact of the COVID-19 Pandemic on Therapeutic Choices in Thrombosis-Hemostasis published on April 15, 2020 [10].

For patients admitted to the hospital on a DOAC, Thachil et al. advise caution as it may have interactions with anti-retroviral drugs and would need closer monitoring in patients with unstable kidney function as the DOAC is likely to accumulate [9]. This was also addressed by Testa et al. in their publication Switch from Oral Anticoagulation to Parenteral Heparin in SARS-CoV-2 Hospitalized Patients on April 15, 2020 [11]. They explained that the reason for replacing oral anticoagulants with parenteral heparin is increased instability of prothrombin time (PT)/INR due to the high variability of vitamin K metabolism, diet, fasting, co-medications, liver impairment and heart failure in patients hospitalized with COVID-19 treated with VKA [11]. Patients on DOACs may also be exposed to over/under treatment caused by pharmacological interferences [11].

On April 17, 2020, Barrett at al. published a comment on the ISTH interim guidelines introduced earlier in March [12]. In their comment, they expressed concerns that in clinical practice patients continue to clot despite being on prophylactic doses of heparin after admission to hospital [12]. Patients tend to occlude their central venous catheters, dialysis filters, and have frequent thrombotic complications such as strokes, pulmonary embolism, deep venous thrombosis, or ischemic limb [12]. High levels of D-dimer and fibrinogen have been previously discussed as signs of hypercoagulable state. Low anti-thrombin levels, however, have not been previously addressed, which render patients more resistant to heparin agents and make prophylactic heparin or LMWH doses likely inadequate [12]. Barrett et al. also point out that survival advantage for patients with COVID-19 who received a prophylactic dose of heparin is clear but may be flawed because the comparison was made against a group of patients who did not receive a prophylactic dose of heparin. They further reiterate that there has never been a disease like COVID-19 that consistently causes thrombotic events in a large number of patients [12]. Barrett et al. state that due to lack of good randomized controlled trials available to guide better anticoagulation management in COVID-19 patients, early initiation of therapeutic anticoagulation with UFH should be considered in hospitalized patients if there are no contraindications or increased risk of bleeding [12]. They also suggest a possible use of anti-thrombin supplementation given the low levels of anti-thrombin in COVID-19 patients [12]. In their article, they make a reference to the British Society for Haemotology recommendations for DIC management with therapeutic anticoagulation using UFH [13]. Of note, in addition to low levels of anti-thrombin in COVID-19 patients that could be contributing to heparin resistance, higher levels of fibrinogen may also be a contributing factor [14]. Barrett et al. suggested that patients who develop pulmonary embolism and are receiving LMWH may be at an increased risk of bleeding if LMWH has to be given with tissue plasminogen activator (tPA), which further supports the use of therapeutic UFH [12]. In addition, renal failure is common in severe cases of COVID-19, making UFH a better choice of anticoagulation [12].

Lastly, while we are trying to establish better control over coagulopathy, it may be of some value to consider an agent that has been used in Japan for the past 30 years to treat DIC and pancreatitis complications. Asakura et al. discussed this agent in their Potential of Heparin and Nafamostat Combination Therapy for COVID-19 article published on April 17, 2020 [15]. Nafamostat is a serine protease inhibitor that inhibits proteolytic enzymes such as thrombin, plasmin, or trypsin [15]. While this may not be an anticoagulant of choice, it may be an agent that potentiates heparin in patients with COVID-19 induced coagulopathy. More studies will need to be done to assess its indication to treat COVID-19 induced coagulopathy. 

## Conclusions

While multiple studies have shown that heparin is beneficial in treating COVID-19-induced coagulopathy, in clinical practice, there have been failures in patients’ response to prophylactic doses when given at the time of admission as well as therapeutic doses when given later in the hospital course. Failure to respond to anticoagulation could be due to low levels of anti-thrombin and high levels of fibrinogen that render patients more resistant to heparin agents potentially making prophylactic doses inadequate. More research is needed to assess if initiating therapeutic doses of heparin earlier as well as administering anti-thrombin supplements may be beneficial. Because of the risk for venous thromboembolism, pulmonary embolism, and renal insufficiency, UFH may be a better choice of anticoagulant in the COVID-19 population when compared to LMWH. In outpatient settings, it may be appropriate to transition qualified patients on VKA to a DOAC to reduce frequency of the needed laboratory monitoring. Lastly, more studies are needed on nafamostat, an anticoagulant frequently used in Japan for DIC in COVID-19 patients, to further assess its efficacy as a potentiate for heparin therapy.
